# Reduced Nitrates/Nitrites and Natural Additives in Organic Fermented Dry‐Cured Sausages: Effects on Microbiological Safety, Quality, and Consumer Acceptance

**DOI:** 10.1111/1750-3841.70985

**Published:** 2026-03-14

**Authors:** Immaculada Argemí‐Armengol, Guillermo Ripoll, Daniel Villalba, Javier Álvarez‐Rodriguez

**Affiliations:** ^1^ Departament of Animal Science Universitat de Lleida Lleida Spain; ^2^ Animal Science Department Centro de Investigación y Tecnología Agroalimentaria Aragón (CITA) Zaragoza Spain; ^3^ Instituto Agroalimentario de Aragón – IA2 (CITA‐Universidad de Zaragoza) Zaragoza Spain; ^4^ Departamento de Producción Animal y Ciencia de los Alimentos, Escuela Politécnica Superior de Huesca, Carretera de Cuarte s/n University of Zaragoza Huesca Spain

## Abstract

This study examines hydroxytyrosol (HXT), a natural antioxidant, as a partial substitute for curing agents in fermented dry‐cured organic sausages. Increasing health concerns regarding nitrates/nitrites, along with EU efforts to reduce their use, highlight the need for alternatives. The effects of partially replacing potassium nitrate or sodium nitrite with HXT were evaluated in terms of physicochemical, microbiological, and sensory properties. Five treatments were produced: C1 (80 mg/kg KNO_3_), HXT100 (40 mg/kg KNO_3_ + 100 mg/kg HXT), HXT300 (40 mg/kg KNO_3_ + 300 mg/kg HXT), C2 (80 mg/kg NaNO_2_), and HXT200 (40 mg/kg KNO_2_ + 200 mg/kg HXT). HXT significantly reduced weight loss and lipid oxidation and accelerated the pH drop during early fermentation, potentially enhancing beneficial microbial activity. Proximate composition (moisture, fat, protein, ash) and water activity were unaffected. Sausages containing HXT appeared whiter and brighter (higher L* values). Residual nitrite declined markedly during fermentation across all treatments. HXT100 and HXT300 showed higher *Listeria monocytogenes* counts than the full‐dose control, raising microbial safety concerns. Consumer sensory evaluations (*n* = 204) favored traditional formulations (C1 and C2) for taste, texture, and overall quality. Interestingly, when informed about the use of natural ingredients, younger and health‐conscious consumers rated all samples more positively. These findings suggest that HXT can serve as a clean‐label antioxidant in cured meats; however, using it as a complete replacement for nitrates/nitrites may compromise safety. A balanced approach is recommended for developing future organic meat products.

**Practical Applications:**

This study demonstrates that hydroxytyrosol (HXT), a natural antioxidant derived from olives, can partially replace nitrates or nitrites in organic fermented dry‐cured sausages. However, complete replacement may compromise food safety. These findings may support the development of cleaner label products with fewer additives and greater health appeal. Nonetheless, consumer sensory evaluations favored traditional formulations in terms of taste, texture, and overall quality.

## 1 Introduction

Curing is an ancient preservation technique used to extend the shelf life of food products. Specific curing processes exhibit regional variations across Europe (Flores 1997). Mediterranean fermented dry‐cured pork sausages are produced from a mixture of lean pork, pork fat, sugar, salt, nitrite and/or nitrate curing salts, and spices (Pérez‐Alvarez et al. 2022). They are typically stuffed into natural casings and subjected to fermentation, curing, and drying processes lasting approximately 70–80 days. These sausages constitute a highly diverse product category, often exhibiting substantial variation in ingredient levels. Differences in NaCl concentration and, consequently, water activity (*a*_w_) arise from variation in initial salt content, fat percentage, and specific drying conditions applied during ripening (Roca and Incze 1990). The maturation rate of fermented sausages is crucial, as it significantly influences overall production time.

Nitrite (E 249–250) and nitrate (E 251–252) salts are well known for providing the characteristic color and flavor of cured meats. Nitrite also inhibits the growth of pathogens such *Clostridium botulinum* and slows the growth of *Listeria monocytogenes* (Shakil et al. 2022; Cammack et al. 1999), although it does not completely prevent it (EFSA 2017; Xi et al. 2011). Nitrite contributes to the formation of stable nitroso‐pigments responsible for the typical red color of cured sausages and hams. It also provides antioxidant activity, improving lipid and protein stability and influencing aroma development (Zhang et al. 2023). Nitrite use is more common in northern Europe, while nitrate is favored in southern Europe due to differences in smoking and curing practices (Olesen et al. 2004).

Nitrite and nitrate ingested from food are rapidly absorbed by the human body and largely excreted as nitrate. Some nitrate is recirculated through salivary glands and converted by oral bacteria to nitrite. Absorbed nitrite can oxidize hemoglobin to methemoglobin, which reduces oxygen transport capacity. This risk is particularly relevant in infants under 1 year of age; therefore, spinach and similar foods are not recommended due to the potential formation of nitrosohemoglobin (“blue baby syndrome”; Brender 2020). Nitrite can also contribute to the formation of nitrosamines, some of which are carcinogenic, mutagenic, or teratogenic (EFSA 2017; Sindelar and Milkowski 2011).

The European Union has recently established new regulatory limits to reduce consumer exposure to N‐nitrosamines while maintaining sufficient protection against pathogenic bacteria (EU Commission 2023; No. 2023/2108). Since October 9, 2025, the maximum permitted levels for ripened sausages decreased from 150 to 80 mg NO_2_^−^/kg for nitrite and from 150 to 90 mg NO_3_^−^/kg for nitrate. Additionally, Regulation (EU) 2021/1165 (2021) permits the use of sodium nitrite (E 250) and potassium nitrate (E 252) in organic foods, each with a maximum ingoing amount of 80 mg.

A variety of plants materials and natural extracts (including celery, rosemary, tea, cloves, basil, oregano, grape seed extract, pine bark, cranberry, and others) have demonstrated antimicrobial activity against foodborne pathogens (Olvera‐Aguirre et al. 2023; Xi et al. 2011). Organic acids are also widely used in the meat industry to inhibit microbial growth, reduce pH and enhance curing performance (Shakil et al. 2022). Before hydroxytyrosol (HXT) was explored, plant extracts, vegetable powders, microbial cultures, and natural antioxidants were the main strategies to replace nitrifying salts in dry fermented sausages (Tahmouzi et al. 2025). Unlike previous strategies that mainly mimicked nitrite's role, HXT adds new value by turning dry fermented sausages into healthier, potentially functional foods. HXT, a phenolic compound derived from the olive trees and olive leaves, has attracted interest as a natural alternative to synthetic antioxidants in dry‐cured sausages due to its ability to prevent lipid and protein oxidation (Chaves‐López et al. 2015; Martínez et al. 2018; Martínez Zamora et al. 2021). HXT also exhibits antimicrobial activity (Medina et al. 2006; Martínez et al. 2018) and aligns with clean‐label and organic product trends. Moreover, its consumption has been associated with cardiovascular health benefits (Quirós‐Fernández et al. 2019, 2022). However, HXT has a strong odor and flavor, making sensory computability and important consideration when incorporating it into meat products.

The hypothesis of this study was that reducing nitrate and nitrite levels when adding HXT to a traditional fermented dry‐cured sausage would not affect its quality or consumer acceptance. To this end, lipid oxidation, microbial growth, physicochemical parameters, and consumer sensory and hedonic responses (in both blind and informed conditions) were examined.

## 2 Materials and Methods

### 2.1 Experimental Design

Five treatments of fermented dry‐cured sausages (Catalan sausage, *llonganissa*) were produced: three containing potassium nitrate (KNO_3_, E 252; powder form) and two containing sodium nitrite (NaNO_2_, E 250; in powder form; from Espècies Teixidor SL, St. Salvador de Guardiola, Spain), at different concentrations. A total of 175 dry‐cured sausages samples (*n* = 30 per treatment) were manufactured. Three treatments combined KNO_3_ with HXT (Mediteanox H10 Olive Fruit extract, Euromed S.A., Mollet del Vallès, Spain; 10% purity, powder form), and two treatments combined NaNO_2_ with HXT, as shown in Table 1. The maximum levels of nitrate (80 mg NO_3_^−^/kg) and nitrite (80 mg NO_2_^−^/kg) were set according to current organic food regulations [Regulation UE 2018/848]. A trial with HXT previously evaluated the possibility of partially replacing synthetic additives (Martínez Zamora et al. 2021) in a similar Spanish fermented sausage. Two contrasting HXT levels (300 mg and 100 mg) were selected in formulations with reduced KNO_3_ to assess dose–response effects on color stability, antioxidant activity, and the microbial reduction of nitrate to nitrite. Using a high and a low dose allowed the identification of both maximal and minimal effective responses. For the NaNO_2_ reduction treatments, a single intermediate dose (200 mg) was selected because nitrite is known to exerts a more direct and potent influence on sensory attributes and microbiological safety (Flores and Toldrà 2021). All five treatments (Table 1) were manufactured on the same day, applying the same processing technology, following a traditional formulation for Catalan fermented dry‐cured sausages (*llonganissa*) and an organic market formulation. The meat used to produce the dry‐cured sausages came from cull sows of Gascon × Duroc crossbreed, organically reared and fed a concentrated feed and forage (from the organic farm “Granja Rotés” in Olius, Lleida, Spain).

**TABLE 1:** Combinations of nitrate and nitrite and hydroxytyrosol in the formulation mixture (mg/kg of raw meat, *n* = 30 per treatment).

Treatments	KNO_3_ (mg/kg)	NaNO_2_ (mg/kg)	HXT (mg/kg)
C1	80	0	0
C2	0	80	0
HXT100	40	0	100
HXT200	0	40	200
HXT300	40	0	300

Abbreviations: HXT, hydroxytyrosol; KNO_3_, potassium nitrate (E 252); NaNO_2_, sodium nitrite (E 250).

C1, control samples with nitrate (80 mg NO_3_^−^/kg); HXT100, samples with nitrate (40 mg NO_3_^−^/kg) and enriched with 100 mg/kg hydroxytyrosol; HXT300, samples with nitrate (40 mg NO_3_^−^/kg) and enriched with 300 mg/kg hydroxytyrosol; C2, control samples with nitrite (80 mg NO_2_^−^/kg); HXT200, samples with nitrite (40 mg NO_2_^−^/kg) and enriched with 200 mg/kg Hydroxytyrosol.

### 2.2 Preparation of Fermented Dry‐Cured Sausage, Processing, and Sampling

The dry‐cured sausages were produced in accordance with European organic production regulations. This traditional product (*llonganissa*) is characterized by its high fat content, which contributes to an extended curing period and results in low acidity (i.e., a comparatively high pH).

Production occurred at the “Dpagès” facilities in Solsona (Lleida, Spain), where the ripening phases were carried out, following the standard industrial production process. Carcasses from three cull sows (over 3 years old) were cut up. For each of five treatments, a total of 15 kg per treatment (15 kg/treatment × 5 treatments = 75 kg of pork) was proportionally weighed from different cuts following a traditional recipe: 29% ham, 16% ribs, 16% shoulder, 10% loin, 10% pork trim, 10% sirloin, 7% belly, and 2% tenderloin. The meat was minced using a stainless‐steel cutting system with 4.5 mm diameter holes (Mainca PM‐98/32). It was then mixed with the remaining ingredients (1.8% fine organic sea salt, 0.4% organic pepper powder, 0.1% organic whole black pepper, and 1% organic cane sugar), as well as the additive at the specified concentrations. The mixture was kneaded for 15 min using a Kneader Mixer (Mainca, RC‐40, Spain), equipped with double paddles designed to mix efficiently without introducing air pockets and create a paste.

The five treatments (15 kg each) were prepared without added starter cultures. Each meat paste, formulated with its respective nitrite/nitrate salt and/or HXT concentrations, was stored for 24 h in a chamber at 3.1 ± 1°C and 52.2 ± 5% relative humidity (RH). The next day, the mixtures were stuffed into natural pork casings (Escorxador Frigorífic d'Avinyó SA) using an automatic stainless‐steel sausage filler (Mainca FC‐12, Spain). Casings were predesalted and washed. Each treatment used a different trussing string color (white, red, green, blue, and mixed blue/red) with numbered labels.

Each sausage weighed approximately 500 g and measured 7–10 cm in diameter and 30–40 cm in length. The 30 sausages per treatment allowed assessment of variability within the curing process: 15 sausages for physical–chemical analyses and 15 for sensory analysis. The design of the experiment prioritizes internal validity by minimizing experimental error through the use of a single, standardized mixture across the five batches. At the same time, external validity is preserved by ensuring that this mixture contains tissue contributions from multiple animals and diverse anatomical sources. After stuffing, the sausages were labeled, individually weighed, and hung on a trolley‐rack in the same chamber for 3 days (3.1 ± 1°C and 52.2 ± 5% RH) to allow controlled fermentation by endogenous microbiota and slight drying of casing. The trolley‐rack was then transferred to an air‐drying chamber at 8–10°C and 76%–83% RH for approximately 2 months to complete fermentation and drying. This temperature and humidity schedule follows traditional Catalan practice, which favors slow fermentation, gradual drying, and the development of characteristic sensory properties. After curing, sausages were vacuum packed and stored at 6°C for 2 months prior to analysis in order to reflect the sensory properties at typical consumption time (often up to 6–12 months).

From each treatment, 200 g of minced meat mixture was sampled on Day 1. Additionally, five sausages were collected at key processing stages: after preripening (Day 10), after fermentation/onset of ripening (Day 28), and at the end of the dry curing (Day 70). Half of each sample (Days 1, 10, 28, and 70) was used for pH and color measurements; the remainder was vacuum packed and stored at −20°C for chemical analyses (proximate composition and lipid oxidation). On Day 70, an additional five sausages per treatment were sampled for nitrite, nitrate, and microbiological analysis. Sensory analysis was conducted on the remaining sausages (15 per treatment) after 2 months of vacuum‐packed storage.

### 2.3 Physical–Chemical Determinations and Proximate Composition of the Sausages During Ripening Process

#### 2.3.1 Weight Loss

Samples (*n* = 20/treatment) were weighed to determine weight losses during ripening on Days 1, 10, 28, and 70. Weight loss was expressed as a percentage of the initial weight (Olivares et al. 2010).

#### 2.3.2 pH

Five sausages from each treatment and sampling day were used to measure pH by inserting a pH meter into the center of the sausages (or into the minced meat mixture on Day 1), as described by ISO 2917 (1999). A pH meter equipped with a spear‐tipped probe (Testo 205, Testo AG, Lenzkirch, Germany) and calibrated with pH 4.0 and 7.0 buffers was used on Days 10, 28, and 70 of ripening.

#### 2.3.3 Color Measurement

Lightness (L*), redness (a*), and yellowness (b*) were determined using a portable colorimeter Minolta CM‐700d spectrophotometer (Konica Minolta Sensing Inc., Osaka, Japan) with standard illuminant D65, zero observer angle and an 8‐mm diameter viewing area, in accordance with the International Commission on Illumination (CIE; Olivares et al. 2010). The color descriptors of fermented dry‐cured sausages were carried out on Days 1, 10, 28, 70 of ripening. Lightness (L*), redness (a*), and yellowness (b*) were reported as the average of two randomly selected readings taken from the inner surface after cutting the sausage in half. Mean values were used for statistical analysis. In the CIELab color space, the hue angle ($h_{ab}$) was calculated as

$\;h_{ab}=\tan^{-1}\left(\frac{b*}{a*}\right)\;\times \;\frac{180}{\pi }$, expressed in degrees. Meanwhile, chroma ($C_{ab}^{*}$) (color intensity, also known as saturation index) was calculated as $C_{ab}^{*}=\sqrt[{}]{{\left(a*\right)}^{2}+{\left(b*\right)}^{2}}$.

In addition, reflectance spectra were measured using in the 360–740 nm spectral range, in accordance with AMSA recommendations. Percentage of metmyoglobin (MMb; AMSA 2012) formation was measured using the following equation:

$$\mathrm{MMb}=\left\{1.395\;-\;\left[\frac{\langle A572\;-\;A730\rangle }{\langle A525\;-\;A730\rangle }\right]\right\}\;\times \;100$$

The cured index (CI) was calculated using the following formula: $\mathrm{CI}\;=\frac{R650\;\mathrm{nm}}{R570\;\mathrm{nm}}$ (Cava and Ladero 2023). All determinations were performed in duplicate on each sausage of a different formulations, with five samples per treatment and day.

#### 2.3.4 Proximate Composition of the Sausages

The proximate composition of the sausages (moisture, protein, fat, ash, collagen, and saturated fatty acids [SFAs]) was determined by near‐infrared transmission spectroscopy with calibration using artificial neural networks and a FoodScan2 Lab Meat Analyser (Foss Electric, Hillerød, Denmark) operating in transmittance mode. Analyses followed AOAC Method 2007.04 for meat and meat products (Anderson et al. 2007) and were performed on Days 1, 10, 28, and 70 of ripening. The method is validated for moisture, fat, and protein according to AOAC, and the calibration parameters are provided in Anderson et al. (2007). Calibration statistics are available in FOSS (2020). Moisture content was expressed in g/100 g, whereas the protein, ash, total fat, SFAs, collagen, and salt contents, were expressed in g/100 g of dry matter. Water activity (*a*_w_) analyses were performed on Days 1, 10, 28, and 70 of the ripening process. Five sausages per treatment were used for each day of the curing period studied. Each sausage was individually chopped and minced in a meat mincer (pig casings were removed beforehand). The meat samples were transferred into the sample dish of NIR equipment of 7.9 cm diameter and a depth of 8.8 mm.

#### 2.3.5 Nitrate and Nitrite Residual Content

The residual nitrate content was determined by liquid chromatographic method (HPLC‐UV) as reported by UNE‐EN 12014‐4 (2016), while the nitrite residual content was carried out by spectrophotometric method PNT‐1‐09 (UV–vis; BOE 1979). The determinations were performed in two analytical replicates on five sausages for each formulation.

#### 2.3.6 Lipid Oxidation

Lipid oxidation was assessed by measuring thiobarbituric acid reactive substances (TBARS) analysis, following a modification of the Rosmini et al. (1996) and Álvarez‐Rodríguez et al. (2018), on Days 1, 10, 28, and 70 of curing of sausages. Five samples were taken from each treatment and each day of the ripening period. The acid extraction method consists of homogenization of meat with trichloroacetic acid. Each sausage was individually chopped and minced in a meat mincer for 60 s. Meat samples (0.5 g; two replicates per sample) were transferred in Pyrex glass tubes, and it was mixed with 15 mL of TBARS solution containing 15% trichloroacetic acid (TCA), 0.375% thiobarbituric acid (TBA) and 0.25 N hydrochloric acid (HCl, 37%). The tubes were incubated at 100°C for 15 min to induce color development. Afterward they were cooled in a freezer by 5 min, followed by centrifuging tubes in Centrifuge 5810 R at 3000 × *g* for 10 min at 4°C to obtain the upper phase (aqueous). The absorbance of the supernatant phase was measured at 540 nm with an iEMS Reader MF spectrophotometer (Labsystems Oy, Helsinki, Finland).

A standard calibration curve was created with increasing concentrations (from 0 to 100 µL) of malonaldehyde (MDA), which was obtained by reaction of 1,1,3,3‐tetraethoxypropane (> 96%, TEP) in TBARS solution. The final conversion of TEP to MDA was accomplished by multiplying the number of µM of TEP equivalent per gram of sample by the molecular weight of MDA. The tubes used for the calibrations, including the blank (with three replicates) and samples (two replicates) to be analyzed, were put through the TBA procedure at the same time. Reaction and detection proceeded as previously described. TBARS values are expressed as milligrams of MDA per kilogram of cured meat (mg MDA/kg).

### 2.4 Microbiological Analysis

The microbiological analysis was conducted in an external laboratory at the Agricultural Technology Institute of Castile and León (ITACyL, Guijuelo‐Salamanca, Spain).

For microbiological determinations, five samples per treatment were analyzed at Day 70 in two replicates. Sausages were sampled by aseptically opening the casings with a sterile lancet and collecting meat from different locations along each sausage. Microbial safety was assessed using the following parameters: *Clostridium botulinum* (ISO/TS 17919:2013), which specifies a horizontal molecular method for detecting clostridia carrying botulinum neurotoxin A, B, E, and F genes, and *Listeria monocytogenes* (ALOA COUNT Method, AES 10/05‐09/06), as described by Rubio et al. (2018). The determination of *L. monocytogenes* was performed by plate counting, with a quantification limit of < 10 CFU/g. The detection of *C. botulinum* based on the presence or absence of botulinum neurotoxins A, B, E, and F, assessed by RT‐PCR. The limit of detection for *C. botulinum* was10 genetic copies/µL for BoNT/A, BoNT/B, and BoNT/F, and 100 genetic copies/µL for BoNT/E.

### 2.5 Sensory Analysis

This study was approved by the Ethics Committee of the University of Lleida, code CERT162 (2025). Participants completed an informed consent form at the beginning of the study. Due to the complexity of the experimental design and food safety considerations related to *L. monocytogenes* levels, the sensory evaluation was simplified and limited to the two control treatments (C1 and C2) and the formulation containing HXT200. Treatments HXT100 and HXT300 were not used in this sensory analysis. Fifteen fermented dry‐cured sausages randomly selected out of three treatments (C1, C2, and HXT200; 45 in total) were used by consumer panels.

#### 2.5.1 Consumer Study and Sensory Evaluation of Fermented Dry‐Cured Sausage Slices

A total of 204 consumers participated in the study. Participants were recruited through advertisements posted on social and classes from the University of Lleida (Spain), and conducted for 1 day, from 9:00 am to 7:00 pm. At recruitment stage, no information about the specific aim of the study was provided (sausage tasting only). The online test focused on identifying the key factors that influence consumers’ choices when purchasing fermented dry‐cured sausages. It also involved a blind and informed product assessment. The volunteers completed a survey through an online questionnaire. Data were collected via a “Google form” obtained by scanning the QR code via mobile phone. The study was conducted using a four‐step online questionnaire consisting of: (1) Consumer profile and behavior (collected data on demographics [age, gender, residence, education, children]) and consumption habits related to cured products; (2) First tasting (blind): participants conducted a blind sensory evaluation of three sausage samples (detailed below); (3) Questions on knowledge and awareness of added nitrates and alternative natural products (the questions asked are listed below); (4) Second tasting (informed): participants performed a second sensory evaluation of the same samples, this time with full information about the treatments (added natural products and nitrates).

Each respondent was asked questions regarding the level of importance that they ascribe to different additive and/or food and/or label factors that influence purchasing arguments. These purchasing motives were assessed using a five‐point Likert scale, in which 1 = none or very little importance, 2 = little importance, 3 = average importance, 4 = quite a lot of importance, and 5 = great importance. Afterward participants performed the first test blind evaluation of the samples.

For the tasting tests (blind and informed, first and second, respectively), the fermented dry‐cured sausages were sliced (2.0 mm thickness) by machine cutting (Romagsa, AF 350 FR‐INGR, Ripollet, Spain) and were identified with a three‐digit code were individually places on plates that were served to consumers. The order of the slices at the first and the second tasting was C1 (101 and 401, respectively), C2 (201 and 501, respectively), and HXT200 (301 and 601, respectively). Between two‐sample slice tasting, the consumers ate toasted bread sticks without salt and drank mineral water. Five attributes were assessed with a 1–9 scale (from least [on the left] to best [on the right] score): color of cured, smell of cured, taste of cured, texture (easy chewing) and overall liking.

After the blind tasting, the second questionnaire focused on participants’ perceptions regarding nitrate/nitrite salts and alternative natural products. Statements were rated on a five‐point Likert scale and included: “I prefer to consume local products, that are grown or produced near where I live”; “I read the labels of the products carefully to know their ingredients, elaboration, contents and others”; “The taste of meals is more important than the ingredients”; “My diet and that of my family is very important to me”; “Given the choice of food products, are the following factors important to you?: (a) quality health care and food safety, (b) origin in organic farming and livestock, and (c) produced locally or in the country where I live.”

### 2.6 Statistical Analysis

The data were analyzed with the JMP Pro 16 version, statistical software (SAS Institute, Cary, NC, USA). Data were subjected to ANOVA and Tukey's HSD test was used as a post hoc test to determine statistically significant differences among means (*p* < 0.05). The sausage was the experimental unit for microbial analysis, nitrate and nitrite residual content, lipid oxidation, proximate composition, and physical–chemical analysis. The data were analyzed with a standard least square model, including a fixed effect of formulation treatment and day of ripening, and also, single interactions. The effect of nitrate/nitrite replacement (C1, C2, or HXT) and information about foods (blind or informed) on sensory attributes was analyzed with a mixed model including formulation treatment and information concern as fixed effects and the consumer as a random effect. Values are presented as least square means and standard error of the mean. The level of significance was set at 0.05.

Consumer segmentation was based on social profile, consumption habits, purchasing intentions regarding cured meat, knowledge about nitrates and alternative natural products, and the two sensory tests of fermented dry‐cured sausages (blind and informed). This was accomplished through hierarchical clustering. This splits the responses to the entire questionnaire into groups based on some measures of dissimilarity. The optimal number of clusters was identified at the highest Cubis Clustering Criterion Contingency tables, and the Pearson test were used to assess the differences between clusters concerning sociodemographic variables, the importance of purchasing attributes, and the sensorial tests. Nonparametric Wilcoxon–Kruskal–Wallis tests with pairwise comparisons were conducted to link consumer clusters with their purchasing drivers in blind and informed sensory tastings.

## 3 Results and Discussion

### 3.1 Physical–Chemical Determinations and Proximate Composition of the Sausages During the Ripening and Curing Process

Table 2 shows the physical–chemical changes (weight losses, *a*_w_, and pH) in fermented sausage samples during the dry‐curing process on different storage days (1, 10, 28, and 70). Potassium nitrate (KNO_3_) and sodium nitrite (NaNO_2_) reduction, as well as HXT substitution, affected weight loss and pH. Higher HXT doses (HXT200 and HXT300) resulted in significantly lower weight loss by Day 70 than the control treatments (C1 and C2) and the lower HXT dose (HXT100). This outcome contrasts with the findings of Martínez Zamora et al. (2021), who reported that incorporating phenolic olive extracts (HXT of synthetic and natural origin at 200 mg/kg) into Spanish‐type dry‐cured sausage (*fuet*) did not affect drying losses. Notably, the nitrite treatment (C2) showed lower initial pH values on Day 1 than the corresponding treatments without nitrite, suggesting that nitrite caused a rapid pH reduction (Premi et al. 2025). During dry‐curing, pH values decreased only until Day 28, which favored the myoglobin oxidation (and thus reduced browning) below 5.5. It should be noted that the pH value in HXT200 decreased slightly faster on Day 10 compared with other treatments. In addition, the higher the concentration of HXT, the lower the pH values, with significantly lower values in HXT200 and HXT300 than in HXT100, suggesting that *Lactobacillus* growth (occurring during the first fermentation phase) may have been slightly promoted (Aquilani et al. 2018; Hospital et al. 2015). It is also plausible that the reduced weight loss observed in HXT200 and HXT300 is partially attributable to their lower pH values, which may enhance water‐holding capacity by promoting stronger protein–water interactions in the meat matrix. Moreover, *a*_w_ was not affected by HXT addition or nitrite salts among treatments. As weight losses increased, *a*_w_ decreased; initial values (0.96) fell to approximately 0.80 in the final product. Similar results have been reported for *fuet* sausages (a thinner Catalan fermented dry‐cured sausage, comparable to the current experiment), which are comparable to those analyzed in the present study (Martínez Zamora et al. 2021).

**TABLE 2:** Physicochemical quality evolution (mean ± SD) of the fermented dry‐cured sausages during ripening process (days of storage).

Treatments						
Parameter	Day	C1	HXT100	HXT300	C2	HXT200
Weight (g)	1	488.7 ± 20.32^a^	452.9 ± 19.11^a^	482.5 ± 19.89^a^	472.0 ± 19.49^a^	479.8 ± 19.89^a^
Cumulative drying losses (%)	10	21.36 ± 0.37^g,z^	21.14 ± 0.35^c,z^	19.73 ± 0.36^gh,z^	21.24 ± 0.36^g,z^	18.60 ± 0.36^h,z^
	28	36.82 ± 0.37^d,y^	38.55 ± 0.35^d,y^	34.68 ± 0.35^e,y^	37.71 ± 0.35^d,y^	32.26 ± 0.32^f,y^
	70	48.99 ± 0.37^a,x^	50.46 ± 0.36^a,x^	46.97 ± 0.36^b,x^	50.40 ± 0.35^a,x^	44.08 ± 0.36^c,x^
*a*_w_	1	0.96 ± 0.006^a,x^	0.96 ± 0.006^a,x^	0.96 ± 0.006^a,x^	0.95 ± 0.006^ab,x^	0.97 ± 0.006^a,x^
	10	0.92 ± 0.006^b,y^	0.93 ± 0.006^ab,y^	0.91 ± 0.006^b,y^	0.92 ± 0.006^b,y^	0.92 ± 0.006^bc,y^
	28	0.91 ± 0.006^b,y^	0.94 ± 0.006^ab,y^	0.92 ± 0.006^b,y^	0.94 ± 0.006^c,y^	0.92 ± 0.006^b,y^
	70	0.79 ± 0.006^c,z^	0.80 ± 0.006^c,z^	0.80 ± 0.006^c,z^	0.79 ± 0.006^c,z^	0.80 ± 0.006^c,z^
pH	1	5.84 ± 0.03^ab,x^	5.79 ± 0.03^ab,y^	5.71 ± 0.03^b,xy^	5.68 ± 0.03^c,y^	5.86 ± 0.03^a,x^
	10	5.68 ± 0.03^c,y^	5.78 ± 0.03^ab,y^	5.79 ± 0.03^ab,y^	5.70 ± 0.03^c,y^	5.45 ± 0.03^c,y^
	28	5.42 ± 0.03^c,z^	5.40 ± 0.03^cd,z^	5.36 ± 0.03^cd,z^	5.37 ± 0.03^cd,z^	5.28 ± 0.03^d,z^
	70	5.41 ± 0.03^cd,z^	5.51 ± 0.03^c,z^	5.38 ± 0.03^d,z^	5.41 ± 0.03^cd,z^	5.33 ± 0.03^d,yz^

C1, control samples with nitrate (80 mg/kg); HXT100, samples with nitrate (40 mg/kg) and enriched with 100 mg/kg hydroxytyrosol; HXT300, samples with nitrate (40 mg/kg) and enriched with 300 mg/kg hydroxytyrosol; C2, control samples with nitrite (80 mg/kg); HXT200, samples with nitrite (40 mg/kg) and enriched with 200 mg/kg hydroxytyrosol; *a*_w_, water activity; ^a–h^Means with different letters in the same day of ripening (row) indicate significant differences (*p* < 0.05); ^x–z^Means with different letters in the same treatment (column) indicate significant differences (*p* < 0.05) between ripening times.

Table 3 shows the CIELab color results after 1, 10, 28, and 70 days of ripening. On Day 70, significant differences among treatments were found only for L*. The HXT200 treatment showed higher L* values, indicating greater lightness compared to C2. A higher L* value may indicate a less intense curing process and lower concentrations of cured pigments. D'Arrigo et al. (2024) similarly found that although red grape pomace could partially replace nitrites in sausages, the typical cured color was not fully achieved. In contrast, no significant differences were observed among treatments in the remaining color the coordinates: a* (redness), b* (yellowness), C* (chroma), *h* (hue), %MMb (metmyoglobin), and CI (cured index). The reddish color (a*) exhibited by different treatments did not differ, despite being one of the most important attributes influencing consumer choices of cured sausages (Martínez Zamora et al. 2021). However, in the groups with KNO_3_ (C1, HXT100, and HXT300) added on Day 0, %MMb (the oxidized form of myoglobin containing ferric iron, Fe^3+^) showed a twofold increase from Day 1 to Day 70, consistent with previous observations (King et al. 2023). This effect was not observed in NaNO_2_ treatments (C2 and HXT200), which also had brine added on Day 0; %MMb values were considerably higher in C2 and HXT200 than in the other treatments on Day 1. Consequently, by the end of ripening period (Day 70), the %MMb levels were similar among treatments. The inherent intensive red color of culled sow meat (Oliveira et al. 2021) likely explains the absence of differences in color coordinates, %MMb, and CI among treatments. Meat from cull sows often shows a more intense red color due to higher myoglobin content, but studies indicate that its color stability is not necessarily greater than meat from younger pigs. In fact, cull sow meat can be more prone to oxidative changes, which may reduce stability over storage. This pronounced pigmentation may have reduced the usual color associated with nitrite reduction or substitution, allowing HXT‐containing sausages to maintain redness levels comparable to those of nitrate‐ and nitrite‐treated controls.

**TABLE 3:** Color attributes (mean ± SD) of the fermented dry‐cured sausages during ripening process (days of storage).

		Treatments				
Parameter	Day	C1	HXT100	HXT300	C2	HXT200
	1	45.0 ± 1.4^ab,x^	44.5 ± 1.4^ab,y^	44.5 ± 1.4^ab,x^	43.2 ± 1.4^b,xy^	46.1 ± 1.4^a,xy^
L*	10	46.1 ± 1.4^a,x^	52.3 ± 1.4^a,x^	45.2 ± 1.4^a,x^	48.9 ± 1.4^a,x^	50.7 ± 1.4^a,x^
	28	44.3 ± 1.4^a,x^	43.2 ± 1.4^a,y^	46.4 ± 1.4^a,x^	45.9 ± 1.4^a,xy^	41.7 ± 1.4^a,y^
	70	41.2 ± 1.4^ab,x^	39.1 ± 1.4^ab,y^	44.1 ± 1.4^ab,x^	37.6 ± 1.4^b,y^	45.4 ± 1.4^a,xy^
	1	9.4 ± 0.82^a,x^	10.9 ± 0.82^a,xy^	9.5 ± 0.82^a,x^	7.6 ± 0.82^a,y^	7.7 ± 0.82^a,y^
a*	10	8.6 ± 0.82^a,x^	7.2 ± 0.82^a,y^	9.4 ± 0.82^a,x^	9.3 ± 0.82^a,xy^	8.4 ± 0.82^a,xy^
	28	11.5 ± 0.82^a,x^	12.2 ± 0.82^a,x^	12.4 ± 0.82^a,x^	12.7 ± 0.82^a,x^	12.1 ± 0.82^a,x^
	70	9.5 ± 0.82^a,x^	8.8 ± 0.82^a,xy^	10.2 ± 0.82^a,x^	10 ± 0.82^a,xy^	9.9 ± 0.82a, xy
	1	9.1 ± 0.64^ab,xy^	10.1 ± 0.64^a,xy^	8.5 ± 0.64^ab,y^	6.8 ± 0.64^b,y^	9.0 ± 0.64^ab,xy^
b*	10	6.5 ± 0.64^b,y^	8.5 ± 0.64^b,y^	6.5 ± 0.64^b,y^	8.9 ± 0.64^b,y^	6.7 ± 0.64^b,y^
	28	12.1 ± 0.64^a,x^	12.1 ± 0.64^a,x^	13.1 ± 0.64^a,x^	12.2 ± 0.64^a,x^	11.6 ± 0.64^a,x^
	70	6.7 ± 0.64^b,y^	6.8 ± 0.64^b,yz^	8.4 ± 0.64^b,y^	6.7 ± 0.64^b,y^	8.2 ± 0.64^b,y^
	1	13.2 ± 0.83^ab,xy^	15.1 ± 0.83^a,y^	12.9 ± 0.83^ab,y^	10.2 ± 0.83^b,y^	11.9 ± 0.83^ab,xy^
C*	10	10.9 ± 0.83^b,y^	11.2 ± 0.83^b,xy^	11.5 ± 0.83^b,y^	13 ± 0.83^b,y^	10.8 ± 0.83^b,y^
	28	16.7 ± 0.83^a,x^	17.2 ± 0.83^a,x^	18 ± 0.83^a,x^	17.7 ± 0.83^a,x^	16.8 ± 0.83^a,x^
	70	11.6 ± 0.83^b,y^	11.1 ± 0.83^b,y^	13.3 ± 0.83^b,y^	12 ± 0.83^b,y^	12.9 ± 0.83^b,xy^
	1	44.5 ± 2.82^a,x^	43.5 ± 2.82^a,x^	42.2 ± 2.82^a,x^	42.1 ± 2.82^a,x^	49 ± 2.82^a,x^
*h*	10	37.9 ± 2.82^ab,x^	50.2 ± 2.82^a,x^	34.8 ± 2.82^b,x^	44.1 ± 2.82^ab,x^	38.2 ± 2.82^ab,x^
	28	46.6 ± 2.82^a,x^	44.6 ± 2.82^a,x^	46.6 ± 2.82^a,x^	43.8 ± 2.82^a,x^	43.8 ± 2.82^a,x^
	70	35.1 ± 2.82^b,x^	37.4 ± 2.82^b,x^	39.5 ± 2.82^b,x^	33.7 ± 2.82^b,x^	39.7 ± 2.82^b,x^
	1	15.7 ± 1.37^b,y^	17.2 ± 1.37^b,y^	15.1 ± 1.37^b,y^	32.4 ± 1.37^a,x^	33.5 ± 1.37^a,x^
%MMb AMSA	10	20.4 ± 1.37^b,xy^	20.0 ± 1.37^b,y^	21.3 ± 1.37^b,xy^	22.7 ± 1.37^b,y^	25.1 ± 1.37^b,y^
	28	27.4 ± 1.37^a,x^	28.1 ± 1.37^a,x^	27.9 ± 1.37^a,x^	28.7 ± 1.37^a,xy^	26.9 ± 1.37^a,xy^
	70	28.5 ± 1.37^a,x^	29.7 ± 1.37^a,x^	28.9 ± 1.37^a,x^	28.8 ± 1.37^a,xy^	27.8 ± 1.37^a,xy^
CI	70	2.5 ± 0.15^a^	2.5 ± 0.15^a^	2.6 ± 0.15^a^	2.8 ± 0.15^a^	2.5 ± 0.15^a^

L*, lightness; a*, redness; b*, yellowness; C*, Chroma, *h*, hue angle; %MMb AMSA, metmyoglobin; CI, cured index = R650 nm/R570 nm; C1, control samples with nitrate (80 mg/kg); HXT100, samples with nitrate (40 mg/kg) and enriched with 100 mg/kg hydroxytyrosol; HXT300, samples with nitrate (40 mg/kg) and enriched with 300 mg/kg hydroxytyrosol; C2, control samples with nitrite (80 mg/kg); HXT200, samples with nitrite (40 mg/kg) and enriched with 200 mg/kg hydroxytyrosol; ^a–c^Means with different letters in the same day of ripening (row) indicate significant differences (*p* < 0.05); ^x–z^Means with different letters in the same treatment (column) indicate significant differences (*p* < 0.05) between ripening times.

As shown in Table 4, moisture, ash, protein, lipid, and SFAs contents were not affected by the treatment at the end of curing, and values were consistent with those previously reported for fermented dry‐cured sausages (Škrlep et al. 2019, 2022; Aquilani et al. 2018). Moisture decreased from 59%–60% on Day 1 to 24%–27% on Day 70, regardless of nitrate salt and HXT concentration. Ash content remained constant (∼3.1%) throughout storage. Protein and lipid contents increased as moisture decreased, reaching 26.3% and 42.1%, respectively, on Day 70, with no differences among treatments. Total SFA content was also similar among treatments (∼11.1%). Although detailed fatty acid profiling (e.g., PUFA composition) was beyond the scope of this study, this represents a limitation that should be addressed in future research. HXT300 sausages showed a slightly higher salt percentage (Day 70) compared with C2. However, the difference (< 0.8%) is small and may reflect low variability within groups.

**TABLE 4:** Proximate composition (mean ± SD) of the fermented dry‐cured sausages during ripening process (days of storage).

		Treatments				
Parameter	Day	C1	HXT100	HXT300	C2	HXT200
Moisture (%)	1	60.2 ± 0.84^ab,w^	59.6 ± 0.97^ab,w^	58.9 ± 0.84^ab,w^	60.8 ± 0.84^a,w^	56.5 ± 0.75^b,w^
	10	51 ± 0.84^c,x^	52 ± 0.97^c,x^	50.6 ± 0.84^c,x^	52.3 ± 0.84^c,x^	48.7 ± 0.75^c,x^
	28	38 ± 0.84^d,y^	37.3 ± 0.84^d,y^	37.5 ± 0.84^d,y^	39.2 ± 0.84^d,y^	38.4 ± 0.75^d,y^
	70	27.4 ± 0.84^e,z^	23.9 ± 0.97^e,z^	27.4 ± 0.84^e,z^	23.9 ± 0.84^e,z^	26.2 ± 0.75^e,z^
Ash (%)	1	3.0 ± 0.24^a,x^	2.7 ± 0.27^a,y^	3.3 ± 0.24^a,x^	2.7 ± 0.24^a,x^	2.4 ± 0.21^a,x^
	10	3.4 ± 0.24^a,x^	3.1 ± 0.27^a,xy^	3.6 ± 0.24^a,x^	3.2 ± 0.24^a,x^	3.4 ± 0.21^a,x^
	28	3.3 ± 0.24^a,x^	4.2 ± 0.27^a,x^	4.0 ± 0.24^a,x^	4.0 ± 0.24^a,x^	3.0 ± 0.21^a,x^
	70	3.2 ± 0.24^a,x^	3.3 ± 0.27^a,xy^	2.8 ± 0.24^a,x^	3.6 ± 0.24^a,x^	2.8 ± 0.21^a,x^
Protein (%)	1	16.9 ± 0.45^d,z^	16.9 ± 0.52^d,z^	16.4 ± 0.45^d,z^	17.2 ± 0.45^d,z^	15.8 ± 0.40^d,z^
	10	21.2 ± 0.45^c,y^	20.8 ± 0.52^c,y^	20.0 ± 0.45^c,y^	20.8 ± 0.45^c,y^	19.8 ± 0.45^c,y^
	28	26.9 ± 0.45^ab,x^	27.7 ± 0.52^a,x^	25.9 ± 0.45^ab,x^	27.4 ± 0.45^a,x^	24.6 ± 0.40^b,x^
	70	26.6 ± 0.45^b,x^	27.2 ± 0.52^b,x^	25.4 ± 0.45^b,x^	27.2 ± 0.45^b,x^	25.0 ± 0.40^b,x^
Lipid (%)	1	17.8 ± 0.76^e,y^	18.9 ± 0.88^e,y^	19.5 ± 0.76^de,y^	17.3 ± 0.76^e,z^	23.3 ± 0.68^d,y^
	10	21.9 ± 0.76^de,y^	21.6 ± 0.88^de,y^	23.4 ± 0.76^de,y^	21.5 ± 0.76^e,y^	25.5 ± 0.68^d,y^
	28	26.7 ± 0.76^bc,x^	27.9 ± 0.88^bc,x^	29.6 ± 0.76^bc,x^	26.6 ± 0.76^c,x^	21.4 ± 0.68^b,x^
	70	39.8 ± 0.76^a,w^	43.2 ± 0.88^a,w^	41.3 ± 0.76^a,w^	42.9 ± 0.76^a,w^	43.2 ± 0.68^a,w^
Collagen (%)	1	1.9 ± 0.22^b,xy^	2.0 ± 0.25^b,y^	1.7 ± 0.22^b,y^	1.3 ± 0.22^b,y^	1.8 ± 0.19^b,y^
	10	0.6 ± 0.22^c,yz^	0.7 ± 0.25^c,y^	0.5 ± 0.22^c,z^	0.8 ± 0.22^c,y^	0.7 ± 0.22^c,z^
	28	1.6 ± 0.22^b,y^	1.4 ± 0.25^b,y^	1.8 ± 0.22^b,y^	1.5 ± 0.22^b,y^	1.8 ± 0.19^bc,y^
	70	2.9 ± 0.22^ab,x^	3.4 ± 0.25^a,x^	2.4 ± 0.22^b,y^	3.8 ± 0.22^a,x^	2.2 ± 0.19^b,y^
Salt (%)	1	2.0 ± 0.11^c,y^	1.9 ± 0.13^c,y^	1.9 ± 0.11^c,y^	1.9 ± 0.11^c,y^	2.0 ± 0.10^c,y^
	10	2.5 ± 0.11^b,xy^	2.4 ± 0.13^b,xy^	2.5 ± 0.11^b,xy^	2.2 ± 0.11^b,xy^	2.6 ± 0.10^b,x^
	28	3.0 ± 0.11^a,x^	2.9 ± 0.13^a,x^	2.9 ± 0.11^a,xy^	2.8 ± 0.11^a,x^	2.6 ± 0.10^a,x^
	70	3.0 ± 0.11^ab,x^	2.4 ± 0.13^ab,xy^	3.1 ± 0.11^a,x^	2.3 ± 0.11^b,xy^	2.8 ± 0.11^ab,x^
Saturated	1	7.4 ± 0.32^bc,y^	8.6 ± 0.37^b,xy^	7.9 ± 0.32^b,y^	6.0 ± 0.32^c,z^	11.2 ± 0.29^a,x^
Fatty acids	10	7.9 ± 0.32^b,y^	7.6 ± 0.37^b,y^	8.3 ± 0.32^b,y^	7.8 ± 0.32^b,y^	9.0 ± 0.29^b,y^
(%)	28	10.1 ± 0.32^a,x^	10.2 ± 0.37^a,x^	10.6 ± 0.32^a,x^	9.9 ± 0.32^a,x^	11.0 ± 0.29^a,x^
	70	10.8 ± 0.32^a,x^	11.1 ± 0.37^a,x^	11.1 ± 0.33^a,x^	11.1 ± 0.33^a,x^	11.4 ± 0.29^a,x^

C1, control samples with nitrate (80 mg/kg); HXT100, samples with nitrate (40 mg/kg) and enriched with 100 mg/kg hydroxytyrosol; HXT300, samples with nitrate (40 mg/kg) and enriched with 300 mg/kg hydroxytyrosol; C2, control samples with nitrite (80 mg/kg); HXT200, samples with nitrite (40 mg/kg) and enriched with 200 mg/kg hydroxytyrosol; ^a–d^Means with different letters in the same day of ripening (row) indicate significant differences (*p* < 0.05); ^w–z^Means with different letters in the same treatment (column) indicate significant differences (*p* < 0.05) between ripening times.

Regarding collagen content, treatment C2 showed a higher level (3.8%) compared with HXT300 and HXT200 treatments (2.4% and 2.2%, respectively). All values fall within the acceptable range for pork fermented dry‐cured sausages (< 4.8 g/100 g dry matter, Spanish Regulation BOE 2014). This indicates that the raw materials used did not contain excessive connective tissue, despite originating from culled sows. Variations may have arisen during the meat mixing. Connective tissue negatively affects the chewiness, of drying does not gelatinize native collagen (Bañón et al. 2010).

Lipid oxidation results are shown in Table 5. Formulations without HXT (C1 and C2) exhibited higher lipid oxidation (0.1–0.2 mg/kg more malondialdehyde) compared with HXT treatments (HXT100–200–300 mg HXT/kg). Importantly, TBARS values (0.3–0.4 mg MDA/kg) remained well below the sensory detection threshold of 0.5 mg/kg (Dunshea et al. 2005), and lower than values reported for other organic dry‐fermented sausages (0.88 mg MDA/kg; Škrlep et al. 2019).

**TABLE 5:** Lipid oxidation (mg MDA/kg of meat) and residual nitrate and nitrite (mg/kg) of the fermented dry‐cured sausages (mean ± SD).

Treatments						
Parameter	Day	C1	HXT100	HXT300	C2	HXT200
mg MDA/kg of meat^1^	28	0.2 ± 0.02^a^	0.2 ± 0.02^a^	0.2 ± 0.02^a^	0.2 ± 0.02^a^	0.2 ± 0.02^a^
	70	0.4 ± 0.02^b^	0.2 ± 0.02^a^	0.2 ± 0.02^a^	0.3 ± 0.02^b^	0.2 ± 0.02^a^
Residual nitrate ion (mg/kg)	70	18.8 ± 1.4^ab^	18.8 ± 1.4^ab^	16 ± 1.4^b^	24.8 ± 1.4^a^	15 ± 1.4^b^
Residual nitrite ion (mg/kg)	70	6.0 ± 0.46^a^	5.7 ± 0.46^a^	5.3 ± 0.46^a^	7.2 ± 0.46^a^	5.9 ± 0.46^a^

C1, control samples with nitrate (80 mg/kg); HXT100, samples with nitrate (40 mg/kg) and enriched with 100 mg/kg hydroxytyrosol; HXT300, samples with nitrate (40 mg/kg) and enriched with 300 mg/kg hydroxytyrosol; C2, control samples with nitrite (80 mg/kg); HXT200, samples with nitrite (40 mg/kg) and enriched with 200 mg/kg hydroxytyrosol. ^a–c^Means within treatment and trait with no common letter differ significantly (*p* < 0.05).

^1^MDA levels did not differ significantly between Day 1 and Day 28 (*p* > 0.10).

Table 5 also shows the residual nitrate (NO_3_^−^ mg/kg) and nitrite (NO_2_^−^ mg/kg) levels of fermented dry‐cured sausage samples at the end of curing period (70 days of storage). Nitrate and nitrite concentrations are expressed as the corresponding ions (NO_3_^−^ and NO_2_^−^), following analytical and regulatory conventions. C2, which had no added NO_3_ but NO_2_^−^ (80 mg/kg), was the treatment with higher (*p* < 0.05) residual NO_3_^−^ content, compared to HXT200 (40 mg NaNO_2_/kg added) and HXT300 (40 mg KNO_3_/kg added). These results would confirm previous findings, observing that nitrite added to meat partially oxidizes (10%–40%) to nitrate over time within meat (Arnau et al. 2013; Honikel 2008), with residual levels of approximately 10–15 mg NO_3_^−^/kg (Nuñez De González et al. 2012). However, no differences in residual NO_2_^−^ concentration were found among the five treatments at the end of curing. The concentration of residual nitrite sharply decreased during fermentation, less than 7 mg/kg, reaching values of approximately 85%–91% of the initial nitrite added to treatments (C2 and HXT200, 80 and 40 mg NO_2_^−^/kg, respectively). These results confirmed the findings of Fernández‐López et al. (2008) and Li et al. (2013), who observed continued nitrite decline during storage.

### 3.2 Microbiological Analysis

As shown in Table 6, after 70 days of ripening, significant differences in *Listeria monocytogenes* counts were observed among the treatments. Specifically, the formulations with a 50% reduction in nitrate (HXT100 and HXT300) exhibited final counts up to 2.5 log CFU/g higher than those in maximum permitted nitrate treatment (C1) and the maximum permitted nitrite treatment (C2) as well as the 50% nitrite reduction treatment (HXT200). These findings confirm the important antimicrobial role of nitrate and nitrite in dry‐fermented sausages. Therefore, reducing the use of these preservatives could result in higher final *L. monocytogenes* counts if the meat batter is contaminated (Hospital et al. 2012; Ricci et al. 2018). However, these findings may raise concerns, as USDA regulations prohibit the use of sodium nitrite and nitrate in natural and organic processed meats (USDA 2005). No *Clostridium botulinum* or toxin production (A, B, E, F) was detected in any treatment at the end of the process.

**TABLE 6:** *Listeria monocytogenes* and *Clostridium botulinum* (mean ± SD) of the fermented dry‐cured sausages at the end of cured process.

Treatments					
	C1	HXT100	HXT300	C2	HXT200
*L. monocytogenes* (log CFU/g)					
Day 70	2.8 ± 0.24^bc^	3.6 ± 0.24^ab^	4.4 ± 0.24^a^	2.0 ± 0.24^c^	1.9 ± 0.24^c^
*Clostridium botulinum* (neurotoxins A, B, E, F)					
Day 70	n.d.	n.d.	n.d.	n.d.	n.d.

C1, control samples with nitrate (80 mg/kg); HXT100, samples with nitrate (40 mg/kg) and enriched with 100 mg/kg hydroxytyrosol; HXT300, samples with nitrate (40 mg/kg) and enriched with 300 mg/kg hydroxytyrosol; C2, control samples with nitrite (80 mg/kg); HXT200, samples with nitrite (40 mg/kg) and enriched with 200 mg/kg hydroxytyrosol. n.d., not detected. ^a–c^Within *L. monocytogenes* counts row, different letter denotes statistical differences across treatments (*p* < 0.05).

### 3.3 Sensory Analysis

Table 7 summarizes the consumer questionnaire results (*n* = 204), including sociodemographic characteristics and lifestyle factors used to define consumer clusters based on blind choices of dry‐cured meat products. Consumers were divided into two clusters according to age and frequency of cured meat consumption. Cluster 1 comprised individuals older than 24 years who consumed cured meat up to twice per week (infrequent consumers), whereas Cluster 2 included individuals younger than 24 years who consumed cured meat 2–4 times/week (frequent consumers; *p* < 0.05). No differences were observed between clusters regarding purchasing attitudes or preferences for labels, additives, or knowledge of chemical substances.

**TABLE 7:** Sociodemographic characteristics, segment profiles in purchase attitudes, and consumer preferences for cured meat products in the blind condition.

		Cluster 1 (%)	Cluster 2 (%)	*p* value
	*n* =	86	118	—
Characteristics of consumers				
Age	< 24 years	72.1	85.6	0.018
Gender	Female	62.8	52.5	0.262
Living area	Rural area (< 2000 inhabitants)	31.4	27.1	0.47
	Medium size area (2000–10,000 inhabitants)	25.6	21.2	
	Urban area (> 10,000 inhabitants)	43.0	51.7	
Have children	Yes	5.8	10.2	0.266
Education	University studies	26.7	25.4	0.832
Frequency of dry‐cured products	3–4 times/week	45.4	63.6	0.027
	1–2 times/week	46.5	28.8	
	< 1 time/week	8.1	7.6	
Purchase attitudes and knowledge of consumers				
Place of purchase of cured meat products	Only supermarket	45.4	35.6	0.456
	Only butchery	8.1	10.2	
	Only directly from producers	0.0	0.9	
	Several	46.5	53.4	
Purchase intention according to the information of product labeling	Ingredients or additives or nutrients	46.5	48.3	0.202
	No attention	44.2	33.1	
	Nutritional only	5.8	11.9	
	All of them	3.5	6.8	
Knowledge and awareness about nitrite added to meat	Yes	47.7	47.5	0.976
Knowledge about nitrite is used to inhibit microbial growth	No	5.8	5.1	0.261
	Yes	33.7	23.7	
	I do not know	60.5	71.2	
Knowledge about nitrite is used to increase the red color of meat	No	7.0	7.6	0.562
	Yes	26.7	20.3	
	I do not know	66.3	72.1	
Knowledge about nitrite is used to prolong the self‐live of meat	No	1.2	0.9	0.729
	Yes	40.7	35.6	
	I do not know	58.1	63.6	
Purchase intention replacing chemical food additives with fruit extracts	Yes	100	100	—

Cluster 1 (%), > 24 years and infrequent consumers. Cluster 2 (%), < 24 years and frequent consumers.

After receiving information about the use of nitrate salts as preservatives and their role in color and flavor development in cured meat production, consumers assessed perceived quality and nutritional value using a 1–5 Likert scale (Table 8). Cluster 2 (< 24 years and high consumers of cured meats) assigned significantly higher scores than Cluster 1 to attributes related to local products (*p* < 0.05), reported having more health‐conscious families (*p* < 0.01), and expressed a stronger affinity for organic farming (*p* < 0.01). These trends are consistent with previous studies showing that consumers’ purchasing decisions are strongly influenced by traditional and local foods associated with natural ingredients, emphasizing authenticity in food marketing (Pieniak et al. 2009). Although sensory traits are fundamental intrinsic drivers of consumer preferences (Tuorila 2007), product perception extends beyond sensory evaluation, as consumers incorporate various additional cues when forming judgments (Köster 2009). Credence attributes, in particular, play a significant role in shaping purchasing decisions (Argemí‐Armengol et al. 2019).

Environmental awareness among younger consumers is also associated with increased ecological behavior (Wierzbiński et al. 2021), although some studies (e.g., Swedish consumers) show that taste remains the primary purchase criterion, with organic production being less influential (Magnusson et al. 2001).

**TABLE 8:** Perceived quality and nutritiousness’ scores (1–5 Likert scale) of cured meat products for each identified cluster in informed condition^1^.

Terms	Cluster 1	Cluster 2	*p* value
I prefer to consume local products, that are grown or produced near where I live.	4.1 ± 0.10	4.4 ± 0.09	0.0127
I read the labels of the products carefully to know their ingredients, elaboration, contents and others.	2.8 ± 0.13	3.1 ± 0.11	0.0931
The taste of meals is more important than the ingredients.	3.2 ± 0.12	3.2 ± 0.10	0.7992
My diet and that of my family is very important to me.	4.3 ± 0.08	4.6 ± 0.07	0.0076
Given the choice of food products, are the following factors important to you?			
Quality health care and food safety	4.5 ± 0.09	4.5 ± 0.08	0.8434
Origin in organic farming and livestock	3.1 ± 0.11	3.6 ± 0.10	0.0041
Produced locally or in the country where I live.	3.9 ± 0.10	4.1 ± 0.09	0.1015

^1^ Mean and standard deviations (SD). Cluster 1, > 24 years and infrequent consumers; Cluster 2, < 24 years and frequent consumers. Responses were collected after participants were informed about additives in processed meats, not prior to the information being provided.

In the sensory evaluation of organic pork dry‐fermented sausages conducted under both blind and informed conditions, significant differences were observed among treatments (C1—nitrate, C2—nitrite, and HXT—hydroxytyrosol) for all attributes (Table 9). These results indicate that both consumer demographics and product information influence sensory perception and preference. Consumers rated the cured color, aroma, taste, and overall acceptability of HXT sausages as less intense than those of C1 and C2 (*p* < 0.001). Regarding texture, HXT sausages were perceived as tougher to chew than C1 (*p* < 0.01).

**TABLE 9:** Mean scores (ranked from 1 to 9, from least to most liking) of sensorial parameters of fermented dry‐curing sausages for different treatments and tasting type.

	Treatment (T)			Taste evaluation type^1^ (TE)			*p* value^3^			Repeatibility^4^
Attributes	C1	C2	HXT	Blind	Informed	SEM^2^	(T)	(TE)	(T x TE)	
Color of cured	7.27^a^	7.43^a^	6.48^b^	6.91^b^	7.21^a^	0.075	< 0.0001	< 0.0001	0.4024	0.35
Smell of cured	7.22^a^	7.20^a^	6.3^b^	6.71^b^	7.1^a^	0.081	< 0.0001	< 0.0001	0.1894	0.33
Taste of cured	6.95^a^	7.15^a^	6.17^b^	6.57^b^	6.94^a^	0.090	< 0.0001	< 0.0001	0.8209	0.32
Texture (easy chewing)	7.33^a^	7.11^ab^	6.9^b^	7.10	7.13	0.094	< 0.0001	0.7098	0.522	0.35
Overall assessment	7.26^a^	7.31^a^	6.51^b^	6.90^b^	7.15^a^	0.080	< 0.0001	0.0002	0.978	0.36

C1, control samples with nitrate (80 mg/kg); HXT100, samples with nitrate (40 mg/kg) and enriched with 100 mg/kg hydroxytyrosol; HXT300, samples with nitrate (40 mg/kg) and enriched with 300 mg/kg hydroxytyrosol; C2, control samples with nitrite (80 mg/kg); HXT200, samples with nitrite (40 mg/kg) and enriched with 200 mg/kg hydroxytyrosol.

^1^ *Llonganissa* tasting was performed before (blind) and after (informed) receiving information on the treatments (nitrates or natural extracts).

^2^ SD, standard deviation.

^3^ *p* value of the effect of treatment, taste evaluation type, and their interactions.

^4^ Repeatability was estimated from the consumers (Vc) and residual (Vr) components of the variance of random effects, using the following formula: *r* = (Vc/(Vc + Vr)).

Significant differences were also observed between blind and informed tasting conditions. Liking scores (1–9 Likert scale) were consistently higher under informed conditions (when participants were aware of nitrite and nitrate substitution) than under blind conditions (*p* < 0.0001) for all treatments. These findings align with previous research indicating that consumers are increasingly concerned about the use of nitrate and nitrite use due to potential health risks, including chemical toxicity, carcinogen formation, and reproductive effects (Sebranek and Bacus 2007). These results suggest that providing information about the use of natural ingredients can positively influence consumer perception and acceptance of dry‐fermented sausages. No significant interactions were observed between treatment and tasting events (*p* > 0.05).

Repeatability values (*r* = 0.32–0.36) indicated moderate but acceptable consistency variability among untrained panel.

**FIGURE 1:**
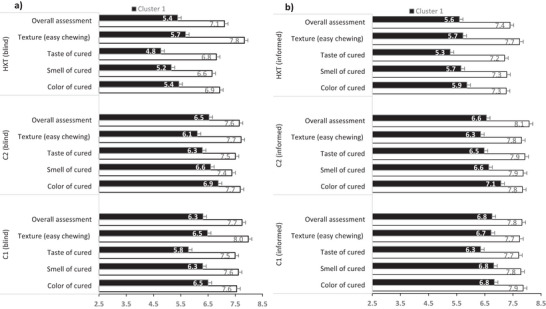
Blind (a) and informed (b) taste clusters for the fermented dry‐cured sausage treatments. Cluster 1: Mores adults (> 24 years of age) and less frequent consumers of dry‐cured sausages (< 2 times/week); Cluster 2 (%): More young adults (< 24 years of age) and more frequent consumers of dry‐cured sausages (2–4 times/week).

Sensory scores (1–9 Likert scale) by Clusters 1 and 2 under both blind and informed conditions confirmed a preference for C1 and C2 over HXT across all attributes (color, aroma, taste, texture, and overall acceptability, *p* < 0.0001; Figure 1). Providing compositional information influenced evaluations: both clusters assigned higher ratings to the same sausage samples when aware that they natural ingredients were used (Table 9 and Figure 1). These findings support previous studies (Hung et al. 2016) showing that consumers favor meat products with reduced nitrite levels and that purchase decisions are influenced by understanding the functional role and perceived health implications of nitrite use.

## 4 Conclusion

This study demonstrates that HXT can effectively serve as a natural antioxidant in fermented dry‐cured organic sausages, improving oxidative stability, promoting a faster early pH decline, and reducing weight loss without altering proximate composition or water activity. However, its partial substitution of nitrate or nitrite revealed important limitations. The objective evaluation of color attributes did not reveal remarkable differences, but consumers scored lower the cured color of the sausages when HXT was added. Treatments containing reduced curing salts (particularly those with KNO_3_ reduction) showed higher *Listeria monocytogenes* counts, underscoring microbiological safety concerns. Consumer testing further indicated that traditional nitrate/nitrite formulations were preferred in terms of flavor, texture, and overall acceptability, despite more favorable evaluations of HXT‐containing sausages when participants were informed about the use of natural ingredients.

Overall, HXT shows promise as a clean‐label antioxidant for organic fermented sausages, but it cannot fully replace nitrates or nitrites without compromising safety and sensory quality. Future work should focus on optimizing combined strategies (such as intermediate curing salt reductions, improved process control, or synergistic use of natural antimicrobials) to achieve safer, consumer‐acceptable, and more sustainable cured meat products.

## Author Contributions

**Immaculada Argemí‐Armengol**: conceptualization, methodology, software, data curation, investigation, visualization, writing – original draft, formal analysis, writing – review and editing. **Guillermo Ripoll**: conceptualization, methodology, investigation, supervision, visualization, writing – review and editing. **Daniel Villalba**: conceptualization, methodology, investigation, supervision, visualization, writing – review and editing. **Javier Álvarez‐Rodriguez**: conceptualization, methodology, data curation, formal analysis, funding acquisition, resources, project administration, visualization, supervision, writing – review and editing, validation.

## Conflicts of Interest

The authors declare no conflicts of interest.

## Data Availability

Data will be made available upon request.
